# Infectious Disease Relational Data Analysis Using String Grammar Non-Euclidean Relational Fuzzy C-Means

**DOI:** 10.3390/ijerph18158153

**Published:** 2021-08-01

**Authors:** Apiwat Budwong, Sansanee Auephanwiriyakul, Nipon Theera-Umpon

**Affiliations:** 1 Department of Computer Engineering, Faculty of Engineering, Graduate School, Chiang Mai University, Chiang Mai 50200, Thailand; apiwat_bouthwong@cmu.ac.th; 2 Department of Computer Engineering, Faculty of Engineering, Excellence Center in Infrastructure Technology and Transportation Engineering, Biomedical Engineering Institute, Chiang Mai University, Chiang Mai 50200, Thailand; 3 Department of Electrical Engineering, Faculty of Engineering, Biomedical Engineering Institute, Chiang Mai University, Chiang Mai 50200, Thailand; nipon.t@cmu.ac.th

**Keywords:** relational data, string grammar non-Euclidean relational fuzzy C-means, Levenshtein distance, dengue fever, influenza, Hepatitis B virus (HBV)

## Abstract

Statistical analysis in infectious diseases is becoming more important, especially in prevention policy development. To achieve that, the epidemiology, a study of the relationship between the occurrence and who/when/where, is needed. In this paper, we develop the string grammar non-Euclidean relational fuzzy C-means (sgNERF-CM) algorithm to determine a relationship inside the data from the age, career, and month viewpoint for all provinces in Thailand for the dengue fever, influenza, and Hepatitis B virus (HBV) infection. The Dunn’s index is used to select the best models because of its ability to identify the compact and well-separated clusters. We compare the results of the sgNERF-CM algorithm with the string grammar relational hard C-means (sgRHCM) algorithm. In addition, their numerical counterparts, i.e., relational hard C-means (RHCM) and non-Euclidean relational fuzzy C-means (NERF-CM) algorithms are also applied in the comparison. We found that the sgNERF-CM algorithm is far better than the numerical counterparts and better than the sgRHCM algorithm in most cases. From the results, we found that the month-based dataset does not help in relationship-finding since the diseases tend to happen all year round. People from different age ranges in different regions in Thailand have different numbers of dengue fever infections. The occupations that have a higher chance to have dengue fever are student and teacher groups from the central, north-east, north, and south regions. Additionally, students in all regions, except the central region, have a high risk of dengue infection. For the influenza dataset, we found that a group of people with the age of more than 1 year to 64 years old has higher number of influenza infections in every province. Most occupations in all regions have a higher risk of infecting the influenza. For the HBV dataset, people in all regions with an age between 10 to 65 years old have a high risk in infecting the disease. In addition, only farmer and general contractor groups in all regions have high chance of infecting HBV as well.

## 1. Introduction

Statistical studies involving infectious diseases have been going on for some time [1]. Some studies model and analyze the development of diseases [1,2,3,4]. Another type of study in infectious diseases is epidemiology, i.e., the study of the frequency of disease and how the frequency differs across groups of people [5,6,7,8,9,10,11,12]. One of the considerations of epidemiology is to look at the relationships inside the data itself. There are many existing methods for analyzing data, including clustering algorithms. However, it has been shown that relational data clustering can find a relationship among data better than regular clustering algorithms [13].

Relational data [13] is described by $R={\left[r_{ij}\right]}_{n\times n}$ where *r_ij_* is a relationship between the *i*th and *j*th objects, and *n* is the number of objects involved. There exist several relational cluster algorithms, e.g., fuzzy non-metric (FNM), assignment prototype (AP) model, relational hard C-means (RHCM), relational fuzzy C-means (RFCM) and non-Euclidean relational fuzzy C-means clustering (NERF-CM) algorithms [13,14,15]. However, these algorithms deal with a numerical feature vector, and the relationship is formed by the pairwise distance between those vectors. Meanwhile, data in healthcare are normally composed of numeric and non-numeric information. Syntactic pattern recognition [16,17,18] is more suitable in this scenario. Although there are a few syntactic clustering algorithms [15,16,18,19,20,21,22,23] that deal with non-numeric datasets, only our relationship clustering algorithm, namely the string grammar relational hard C-means (sgRHCM) algorithm [24], can deal with non-numeric relationship datasets. Since there is normally an uncertainty in a dataset, it would be better to use string grammar relationship fuzzy clustering to cope with the problem. Therefore, in this paper, we introduce a string grammar non-Euclidean relationship fuzzy C-means (sgNERF-CM) algorithm. This algorithm is an extended version of its numeric counterpart NERF-CM algorithm.

In Thailand, reports have been published on the occurrence of infectious diseases [25]. There has been no report on the relationship between province and the occurrence of disease based on age, career, and month. In addition, one might want to know whether there is any relationship between different provinces in terms of the number of infections. However, the collected raw data does not provide this information directly. It does not show clusters based on the relationship between provinces, either. One might use the numeric clustering algorithm to find the clusters of similar province characteristics based on the occurrences of a disease, but the result of that numeric clustering algorithm cannot cluster based on disease occurrence relationship among provinces. Moreover, one is unable to use numeric relational cluster algorithms directly if the dataset does not contain only numeric values. In that case, the use of string grammar relationship clustering might be more appropriate. When we find the clusters based on the disease occurrence relationship of provinces, this might help the country to formulate a good prevention policy. To formulate a good prevention policy, we need to study the epidemiology of these infectious diseases. In this paper, we study dengue fever, influenza, and Hepatitis B virus (HBV) infection. Therefore, we will use our sgNERF-CM algorithm in analysis of these health datasets to see if there is any relationship between province and the occurrence of the diseases based on age, career, and month on the three abovementioned diseases. Therefore, the contribution of the paper is two-fold. First, from a technical perspective, a new algorithm, namely the sgNERF-CM algorithm, is developed. Secondly, from an application perspective, the new sgNERF-CM algorithm is applied in real-world health datasets containing string grammar data, not numeric data.

## 2. String Grammar Non-Euclidean Relational Fuzzy C-Means (sgNERF-CM) Algorithm

We will briefly describe the string grammar non-Euclidean relational fuzzy C-means (sgNERF-CM) algorithm here. Let **S** = {*s*_1_, *s*_2_, …, *s_N_*} be a set of *N* strings [18], each of which is a sequence of symbols (primitives). Suppose *s_k_* = (*x*_1_*x*_2_…*x_l_*), a string with length *l*, where each *x_i_* is a member of a set of defined symbols or primitives ($x_{i}\in \Sigma \;\mathrm{for}\;i=1,\ldots ,l$). The relationship *r_ij_* between input strings *s_i_* and *s_j_* is computed using the Levenshtein distance *Lev*(*s_i_*, *s_j_*) [18] (the smallest number of transformations needed to derive one string from another). The spread transformation parameter (*β*) is used to convert non-Euclidean dissimilarity relationship data into Euclidean dissimilarity data. This transformation is designed to prevent a failure from using non-Euclidean dissimilarity relationship data [14]. The sgNERF-CM algorithm is shown below.

**Store:** Relation matrix R = [*r_ij_*]*_N_*_×__*N*_, where *r_ij_* = *Lev(s_i_, s_j_)*.

**Initial:** *β* = 0, *m* = 1.5, U^(0)^ = [*u_ij_*]*_C_*_×__*N*_ ∈ M*_fCV_*, 1 < *C* < *N* and ε = 10^−3^ where M*_fCN_* is a fuzzy partition matrix [15], *m* is a fuzzifier, and *uik* is the membership value of the *k*th object in the *i*th cluster.

**Do** {

  Update prototype (*v_i_*):

(1) $$v_{i}=\frac{{\left(u_{i1}^{m},u_{i2}^{m},\ldots ,u_{iN}^{m}\right)}^{T}}{\sum_{k=1}^{N}u_{ik}^{m}}\;\mathrm{for}\;1\;\leq \;i\leq \;C$$

  Calculate distance [5]:

(2) $$d_{ik}={\left(R_{\beta }v_{i}\right)}_{k}-\frac{\left(v_{i}^{T}R_{\beta }v_{i}\right)}{2}\;\mathrm{for}\;1\;\leq \;i\leq \;C\;\mathrm{and}\;1\;\leq \;k\leq \;N$$

If *d_ik_* < 0 for any *i* and *k*, then

  Calculate

(3) $$\Delta \beta =\max\left\{-2\times \frac{d_{ik}}{\left({\left\|v_{i}-e_{k}\right\|}^{2}\right)}\right\}$$

  Update

(4) $$d_{ik}=d_{ik}+\left(\frac{\Delta \beta }{2}\right)\times {\left\|v_{i}-e_{k}\right\|}^{2}$$

  Update

(5) β=β+Δβ

If *d_ik_* > 0 for all *i*

  Update membership value:

(6) uik=1∑j=1C(dikdjk)1(m−1)

  Else

(7) $$u_{ik}=0$$

if *d_ik_* > 0, *u_ik_* ∈ [0,1], and $\sum_{j=1}^{C}u_{jk}=1$

  } **Until**

(8) $$\left(\left\|U^{t-1}-U^{t}\right\|\leq \varepsilon \right)$$

To check the cluster validity after the algorithm converges, we compute the compactness and separation of clusters using Dunn’s validity index [26,27] which is a standard cluster validity measure to show the goodness of the clustering result as follows:

(9) $$D=\underset{1\leq i\leq k}{\min}\left(\underset{i+1\leq j\leq k}{\min}\left(\frac{dist\left(c_{i},c_{j}\right)}{\underset{1\leq l\leq k}{\max}diam\left(c_{l}\right)}\right)\right)$$

where *dist*(*c_i_*,*c_j_*) is the distance between clusters *c_i_* and *c_j_* and computed as:

(10) $$dist\left(c_{i},c_{j}\right)=\underset{s_{k}\in c_{i},s_{l}\in c_{j}}{\min}Lev\left(s_{k},s_{l}\right)$$

*diam*(*c_j_*) is the diameter (maximum pairwise distance of strings in a cluster) of cluster *c_j_* and computed as:

(11) $$diam\left(c_{i}\right)=\underset{s_{k},s_{l}\in c_{i}}{\max}Lev\left(s_{k},s_{l}\right)$$

The nature of Dunn’s index is that the larger value, the better the resulting clusters. However, one might wonder why Dunn’s index is used to evaluate the cluster validity in this case, when there are several existing cluster validity methods. The reason is that this index exists simply to calculate and can be easily applied to a string grammar clustering. Additionally, there is, to date, no cluster validity measure in the case of string grammar clustering in the literature.

To assign a test string (*s_t_*) into a cluster, compute

*s_t_* is in the *i*th cluster if *d_it_* < *d_jt_* for *j* ≠ *i* (12)

where [5]

(13) $$d_{it}=-\left(\frac{\alpha }{2}\right)\sum_{p=1}^{N}\sum_{q=1}^{N}u_{ip}u_{iq}\left[-r_{pt}-r_{qt}+r_{pq}\right]$$

with

(14) $$\alpha ={\left(\frac{1}{\sum_{i=1}^{N}u_{it}}\right)}^{2}$$

## 3. System Description

**Figure 1:**
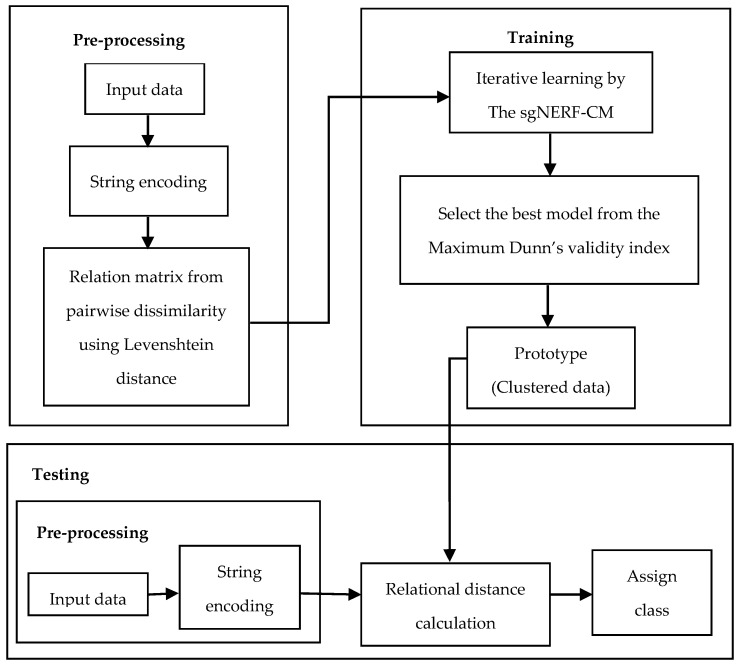
System description.

The system used in this research is shown in Figure 1. Each sample datum is encoded into a string sequence (*s_i_*). Then, the relational matrix between all string sequences is compute using the Levenshtein distance *Lev*(*s_i_*, *s_j_*) [18]. The sgNERF-CM is iteratively computed until it converges. 

The final clusters, based on the disease occurrence relationship of provinces, are produced. To find which cluster belongs to which, based on the relation of each province, we encode that sample into a string sequence. Then, the relationship distance in Equation (2) is computed. The test sample is assigned to the closest cluster.

## 4. Simulation Results

The dengue fever, influenza, and HBV datasets were collected by the Bureau of Epidemiology, Department of Disease Control, Ministry of Public Health, Thailand (http://www.boe.moph.go.th/boedb/surdata/) (accessed on 23 March 2020). These datasets are the reports of the number of suspected infections based on different categories, i.e., the number of infected people arranged by age, career, and month in each province in Thailand. These reports are collected by the provincial public health office of each province, government hospital, and health center. Although the age and career categories are not as good as other categories in health development, we still implement our algorithm using these categories because it might help the policymaker look at the influence of age or career in infection. We split datasets into training and blind test datasets. The detail is as follows. The training dataset for dengue fever is from 2010 and 2012 to 2018, whereas that for influenza is from 2006 to 2018. The report of HBV from 2006 to 2018 is used as a training dataset. In the training process, the algorithm is applied several times for each parameter setting. The parameter setting is selected by randomization method. After the algorithm converges, provinces with similar number of occurrences are grouped into the same cluster. Then, the best model is selected to be used in the cluster assignment of the blind test dataset. The blind test dataset in each disease and each category is from 2019. Table 1, Table 2 and Table 3 show examples of input data in each category from the dengue fever dataset. Please note that we intentionally selected these examples to show the variety of data in each category.

We then convert the data into string data by concatenating the number in each field with commas, as shown in Table 4. For example, suppose that the 1st–4th numbers are 30, 4, 5, and 100, then the concatenated string will be 30, 4, 5, and 100. We train our algorithm on the randomized data from the training dataset. After we select the results with the highest Dunn’s index, we test that model on the blind test dataset. The numbers of training and blind test datasets for all datasets are shown in Table 5.

In the experiment, the number of clusters varies from 2 to 10. To show the ability of the sgNERF-CM algorithm, we also implement the sgRHCM algorithm [24] and their numerical counterparts, i.e., the non-Euclidean relational fuzzy c-means clustering (NERF-CM) algorithm [14] and the relational hard C-means (RHCM) algorithm [15]. The best results of age, career, and month categories are shown in Table 6, Table 7 and Table 8, respectively. We can see that Dunn’s indices for the sgNERF-CM and sgRHCM algorithms are better than their numerical counterparts in all the experiments. The index for the sgNERF-CM algorithm is comparable or better than that for the sgRHCM algorithm in all the experiments.

Since there are many experiment results, we only select the best results and illustrate them in the following figures with a color map of clusters. This is the most concise and understandable way to show the clustering result). Some of the example samples in each cluster are shown in the following tables.

**Figure 2:**
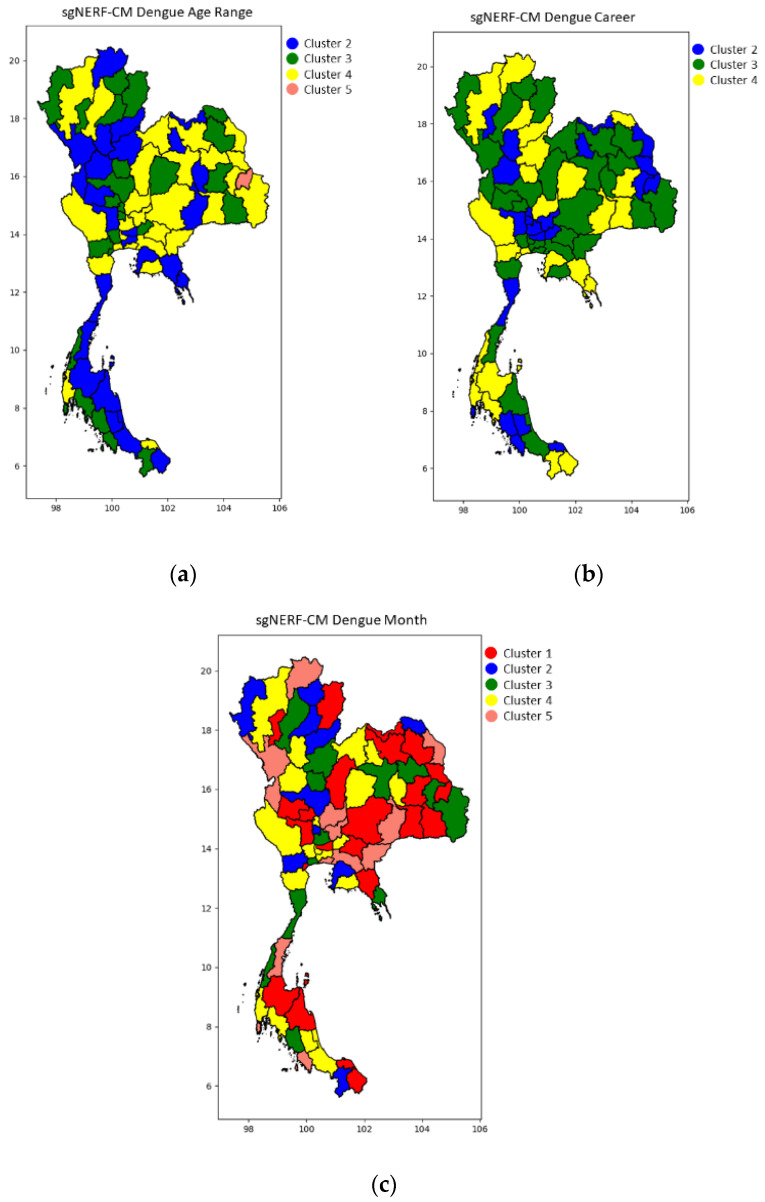
The blind test results for the dengue fever dataset for (**a**) age range, (**b**) career, and (**c**) month category using the best models from sgNERF-CM algorithm.

**Figure 3:**
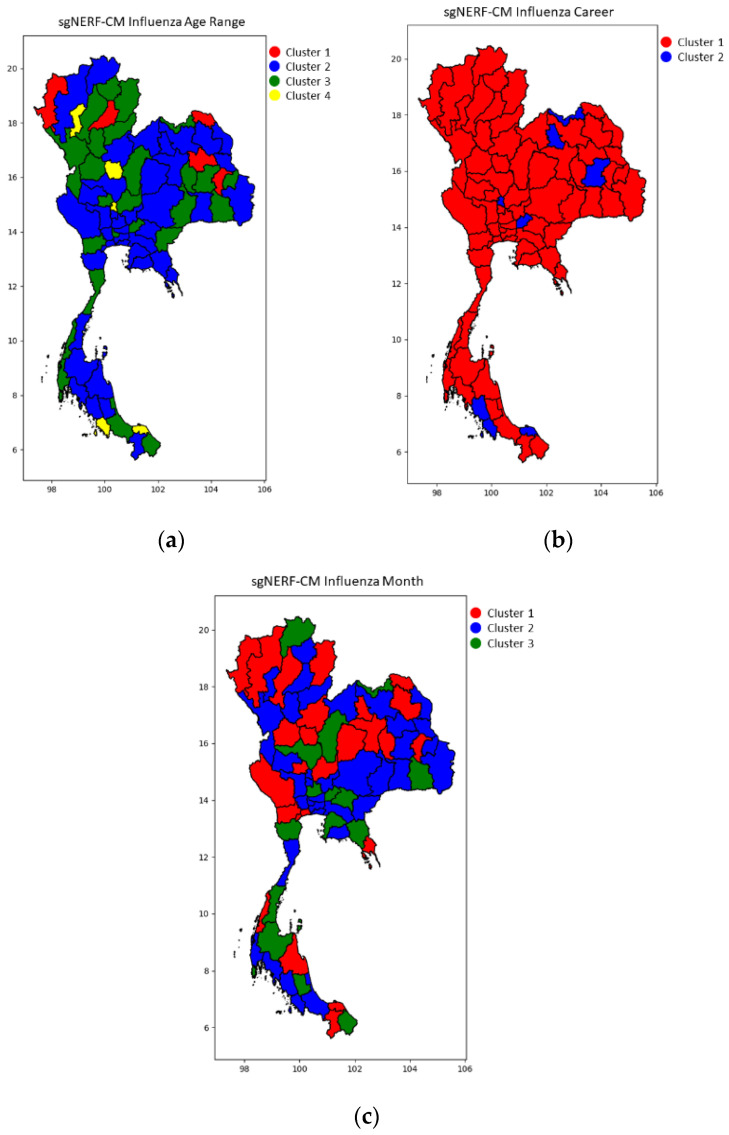
The blind test results for the influenza dataset for (**a**) age range, (**b**) career, and (**c**) month category using the best models from sgNERF-CM algorithm.

**Figure 4:**
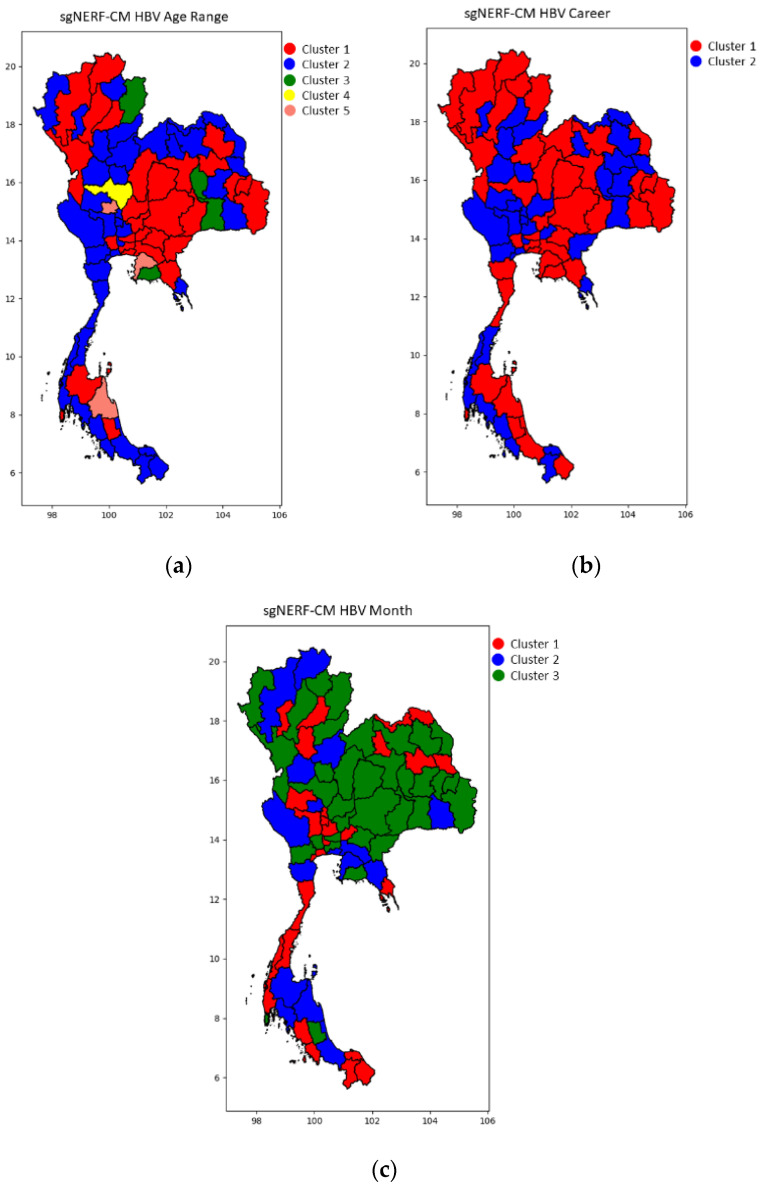
The blind test results for the HBV dataset for (**a**) age range, (**b**) career, and (**c**) month category using the best models from sgNERF-CM algorithm.

Figure 2, Figure 3 and Figure 4 show the blind test clustering results using the best models from the sgNERF-CM algorithm for the dengue fever, influenza, and HBV datasets, respectively. The provinces that are grouped into the same cluster based on their relationship of the number of the disease occurrences in each category are shown in the same colors in the figures. The figures show that the sgNERF-CM can group provinces based on their relationship to the number of disease occurrences. This is also shown in the following discussion.

The sgNERF-CM algorithm can group provinces if there is any relationship based on each specified category. To show this ability, for the dengue fever dataset, examples of the training samples and blind test samples in each group are shown in Table 9, Table 10 and Table 11. Examples of clustering results for the influenza dataset are shown in Table 12, Table 13 and Table 14. Finally, those for the HBV dataset are shown in Table 15, Table 16 and Table 17. From Table 9, we can see that in clusters 2 and 4, a group of people in the north, central, east, north-east, and south regions aged between 10 and 24 years old has a higher number of dengue fever infections, whereas in clusters 3 and 5, a group of people in the region of north-east, central, and south aged between 10 and 14 years old has a higher number of dengue fever infections.

From Table 10, we can see that student and teacher groups have a higher number of dengue fever infections in the 3rd cluster (central, north-east, north, and south region). In the 2nd cluster (central, north-east, north, and south region), the student group has a higher number of dengue fever infections. However, the number of infections in the student group in the 3rd cluster is higher than that in the 2nd cluster. In the 4th cluster, only the student group in all regions except for the central region has a higher number of infections. From this category, we can see that the student group has a chance of being infected by dengue fever more than other occupations. Hence, in this case, a prevention policy can be directed to students in schools, e.g., allocating budget to schools for this particular prevention, providing knowledge to students, giving a recommendation for schools to clean mosquito-breeding habitats, etc. From Table 8, in only the 2nd cluster from the north, north-east, west, and south regions, the number of dengue infections is low. In the 1st cluster (central, north-east, east, and south regions), the number of infections in all 12 months is high. In this case, we can see that there will be a case of infection in every month of year for all regions. The prevention policy should be applied all year round for those abovementioned regions. This can be done in the form of a recommendation to clean mosquito-breeding habitats regularly, promotion of awareness of the disease at all times, etc.

From Table 12, we can see that the 2nd and 3rd clusters behave similarly, i.e., a group of people aged 1 to 64 years old has a higher number of influenza infections in every province. However, in the 4th clusters, only 4 provinces have a lower number of infections. From Table 13, we can see from the blind test examples that the student group in all regions has a higher number of influenza infections in the 1st cluster. In the 2nd cluster from the training examples, only the student and unknown groups have a higher number of influenza infections. However, when we look at the blind test results, we can see that most of the samples are grouped into the 1st cluster. This might be because most of the occupations have a high number of infections. Hence, the generated strings are more related to most of the samples in the 1st cluster than the 2nd cluster. From Table 14, we can see that the number of infections is high in all the clusters (covering all regions) in all 12 months of the year, meaning that the influenza prevention policy should be implemented in all regions. The policy can be executed in the form of a screening and isolation system, and a recommendation or promotion of using sanitary masks in all regions. Additionally, health personnel should emphasize these policies among students and unknown groups.

From Table 15, we can see that in all clusters, there are higher numbers of HBV infections in a group of people in all regions aged 10 to 65 years old. From Table 16, we can see that the farmer and general contractor groups in all regions have a high number of HBV infections in the 1st cluster. However, in the 2nd cluster, only the general contractor group in all regions has high numbers of HBV infections. To develop a prevention policy, these two occupations should be focused on more than other occupations. Promotion of disease awareness and vaccination of people, especially those in these two occupations, should be embedded into health policy. From Table 17, the month information is not useful. This is because when we look at the 3rd cluster (all regions), the number of infections is low in every month, whereas in the 2nd cluster (all regions), the numbers are high in every month.

## 5. Conclusions

To develop health policy, especially in infectious diseases, health data analysis is becoming increasingly important. Epidemiology is the study of finding relationships between occurrences of a disease and other environmental factors (who, when, and where). To analyze infectious disease datasets, we developed the string grammar non-Euclidean relational fuzzy C-means (sgNERF-CM) algorithm to find relationships inside the data from the age, career, and month viewpoints for all provinces in Thailand for dengue fever, influenza, and HBV infection. The input datasets are the reports of the disease occurrences arranged by age, career, and month in each province in Thailand. The developed algorithm is implemented to group provinces based on their relationship of disease occurrences in each category. The cluster results provide additional information to aid health personnel or policymakers to see similarities in each group. This similarity will ultimately help in the development of health policy in the future.

To show the sgNERF-CM algorithm’s performance and ability to cope with uncertain data, we compare the results with the string grammar relational hard C-means (sgRHCM), the relational hard C-means (RHCM), and the non-Euclidean relational fuzzy C-means (NERF-CM) algorithms. The results show that the sgNERF-CM algorithm is better than the sgRHCM algorithm in most cases, and better than the numerical algorithms in all cases. We selected the best sgNERF-CM models from the one yielding the highest Dunn’s index because it indicated the most compact and best-separated clusters. In the blind test process, we found that people from different age ranges in different regions in Thailand have different numbers of dengue fever infections. Student and teacher groups from central, north-east, north, and south regions have higher chances of being infected by dengue fever. Additionally, the student group in all regions except for the central region has a high risk of dengue infection. In every month, people in the central, north-east, east, and south regions should be made aware of the prevention of the dengue fever. For the influenza dataset, we found that a group of people aged 1 to 64 years old has a higher number of influenza infections in every province. Most occupations in all regions have a higher risk of influenza infection. It is not surprising that infection of influenza in all regions happens all year round. For the HBV dataset, people in all regions aged between 10 and 65 have a high risk of disease infection. In addition, only the farmer and general contractor groups in all regions have high chance of contracting the disease as well. Again, it is not surprising that the number of infections by month does not contain specific information, since the infections can happen all year round.

This paper provides information extracted from the collected infectious disease data. We hope that this information will be beneficial in the development of prevention policy. For future work, we plan to apply our sgNERF-CM algorithm to extract useful information for the other diseases. Additionally, we can further use the cluster results to predict disease development in a given region, age, or occupation.

## Figures and Tables

**Table 1:** Age category report from the dengue fever dataset.

Year	Province	Under 28 Days	Under 1 Year	1 Years and Over	2 Years and Over	3 Years and Over	4 Years and Over	5 Years and Over	6 Years and Over	7–9 Years	10–14 Years	15–24 Years	25–34 Years	35–44 Years	45–54 Years	55–64 Years	Over 65 Years	Unknown
2018	Chiang Mai	0	4	4	9	10	8	10	13	51	117	264	268	162	71	73	33	0
2018	Narathiwat	0	3	4	4	11	7	11	13	41	53	65	61	37	17	19	9	0
2017	Chon Buri	0	10	17	8	17	6	17	15	64	145	153	85	46	19	7	5	0
2017	Samut Prakan	0	8	11	18	14	18	17	29	112	192	213	79	47	25	11	6	0
2016	Sukhothai	0	0	2	1	5	8	9	17	31	53	65	21	9	6	4	0	0
2016	Nakhon Sawan	0	7	4	9	7	10	8	12	52	69	125	55	25	23	9	15	0
2015	Uthai Thani	0	3	4	7	15	17	17	32	124	285	324	174	100	44	37	25	0
2015	Bangkok	0	65	87	108	128	129	152	192	798	1711	3338	2520	1668	989	576	277	0
2014	Tak	0	2	2	14	9	10	16	15	52	99	87	35	28	16	5	2	0
2014	Rayong	0	1	5	8	4	9	11	17	36	71	116	80	53	26	14	6	0

**Table 2:** Career category report from the dengue fever dataset.

Year	Province	Farmers	Public Servant	General Contractor	Merchant	Housekeeper	Student	Soldier	Fisherman	Teacher	Other	Unknow	Herdsman	Priest	Special Occupation	Public Health Personnel
2018	Chiang Mai	57	9	411	60	31	331	1	0	6	57	99	0	17	8	10
2018	Tak	341	7	895	77	62	793	11	0	5	146	252	0	12	0	1
2017	Chon Buri	5	1	42	8	6	183	2	0	1	0	37	0	0	0	1
2017	Samut Prakan	17	5	81	10	7	214	1	2	1	3	45	0	0	0	0
2016	Kalasin	57	8	142	7	8	303	1	0	2	3	85	0	9	0	1
2016	Chaiyaphum	58	5	98	6	18	644	5	0	1	194	207	0	0	0	1
2015	Phangnga	140	3	42	3	2	877	7	0	1	1	154	0	2	0	3
2015	Bangkok	57	0	21	5	12	354	0	0	1	20	65	0	3	0	5
2014	Nong Khai	198	6	276	44	30	696	4	2	7	21	76	0	3	0	3
2014	Buri Ram	100	40	113	8	22	106	5	0	4	9	61	0	1	8	61

**Table 3:** Month category report from the dengue fever dataset.

Year	Province	Jan	Feb	Mar	Apr	May	Jun	Jul	Aug	Sep	Oct	Nov	Dec
2018	Trat	6	9	16	15	31	56	71	34	53	38	36	21
2018	Khon Kaen	5	5	6	9	64	132	138	143	120	67	83	80
2017	Samut Sakhon	75	48	36	19	20	26	51	60	42	40	66	26
2017	Suphan Buri	29	38	37	18	5	15	19	25	34	30	32	5
2016	Tak	26	18	20	19	19	31	58	63	43	49	39	17
2016	Uttaradit	13	6	22	11	12	12	37	46	36	7	6	1
2014	Narathiwat	34	19	3	6	13	15	44	75	67	66	48	25
2014	Phatthalung	36	42	29	26	16	69	39	50	40	69	59	48
2013	Bangkok	575	282	292	210	271	0	0	0	0	0	0	0

**Table 4:** Example of generated string sequences.

Disease	Category	Year	Province	String Grammar
Dengue fever	Age Range	2017	Roi Et	2, 12, 15, 28, 25, 49, 45, 75, 350, 735, 555, 129, 61, 38, 29, 16, 0
	Monthly	2018	Ranong	5, 2, 52, 4, 4, 89, 2, 3, 0, 1, 7, 0, 0, 0, 2
	Career	2013	Trat	15, 15, 48, 58, 56, 131, 91, 43, 24, 9, 9, 5
Influenza	Age Range	2018	Phichit	0, 37, 49, 57, 60, 51, 56, 41, 79, 99, 110, 115, 98, 95, 74, 74, 0
	Monthly	2016	Loei	81, 6, 38, 0, 0, 101, 7, 0, 1, 2, 214, 0, 1, 0, 1
	Career	2014	Bangkok	1440, 4216, 3629, 1003, 572, 697, 1055, 1343, 1822, 1298, 1852, 1395
HBV	Age Range	2011	Buri Ram	0, 0, 0, 1, 0, 0, 0, 0, 1, 6, 31, 56, 86, 82, 62, 24, 0
	Monthly	2008	Krabi	4, 0, 11, 0, 2, 2, 0, 0, 0, 1, 1, 0, 0, 0, 0
	Career	2018	Phayao	10, 8, 13, 3, 13, 5, 2, 10, 10, 13, 11, 13

**Table 5:** Number of training and blind test datasets.

Report Categories	Dengue Fever		Influenza		HBV	
	Training	Blind Test	Training	Blind Test	Training	Blind Test
Age Range	487	76	791	76	791	76
Monthly	487	76	791	76	791	76
Career	487	76	730	76	730	76

**Table 6:** The best result from age category.

Data Set	sgNERF-CM		sgRHCM		NERF-CM		RHCM	
	(No. Train, No. of Clusters)	Dunn’s Index	(No. Train, No. of Clusters)	Dunn’s Index	(No. Train, No. of Clusters)	Dunn’s Index	(No. Train, No. of Clusters)	Dunn’s Index
Dengue fever	(3rd, 5)	0.317	(3rd, 6)	0.317	(5th, 2)	0.016	(3rd, 2)	0.013
Influenza	(5th, 4)	0.250	(5th, 7)	0.225	(4th, 6)	0.006	(3rd, 4)	0.013
HBV	(1st, 6)	0.114	(3rd, 3)	0.114	(1st, 2)	0.031	(1st, 2)	0.032

**Table 7:** The best result from career category.

Data Set	sgNERF-CM		sgRHCM		NERF-CM		RHCM	
	(No. Train, No. of Clusters)	Dunn’s Index	(No. Train, No. of Clusters)	Dunn’s Index	(No. Train, No. of Clusters)	Dunn’s Index	(No. Train, No. of Clusters)	Dunn’s Index
Dengue fever	(4th, 5)	0.241	(3rd, 3)	0.232	(3rd, 2)	0.009	(5th, 3)	0.031
Influenza	(1st, 3)	0.214	(1st, 2)	0.214	(4th, 4)	0.003	(5th, 8)	0.005
HBV	(4th, 2)	0.167	(3rd, 2)	0.139	(4th, 2)	0.019	(3rd, 2)	0.013

**Table 8:** The best result from month category.

Data Set	sgNERF-CM		sgRHCM		NERF-CM		RHCM	
	(No. Train, No. of Clusters)	Dunn’s Index	(No. Train, No. of Clusters)	Dunn’s Index	(No. Train, No. of Clusters)	Dunn’s Index	(No. Train, No. of Clusters)	Dunn’s Index
Dengue fever	(3rd, 5)	0.315	(3rd, 8)	0.308	(3rd, 2)	0.010	(5th, 3)	0.031
Influenza	(5th, 3)	0.209	(3rd, 5)	0.222	(5th, 2)	0.005	(5th, 2)	0.006
HBV	(5th, 3)	0.190	(5th, 5)	0.195	(1st, 2)	0.053	(5th, 3)	0.031

**Table 9:** The example of clustering results from the selected models for the dengue fever dataset for the age range category.

Data	Cluster	Province	(<28 Days, <1 Year, 1+, 2+, 3+, 4+, 5+, 6+, 7–9, 10–14, 15–24, 25–34, 35–44, 45–54, 55–64, 65+, Unknown)
Training	1	2561, Phichit	0, 5, 11, 5, 11, 18, 15, 29, 67, 209, 237, 104, 50, 32, 20, 8, 0
	1	2556, Nonthaburi	0, 2, 8, 8, 13, 13, 12, 15, 53, 126, 198, 84, 59, 35, 19, 9, 0
Blind Test	1	No data are assigned	
Training	2	2561, Chiang Rai	0, 22, 25, 32, 42, 55, 59, 47, 159, 323, 538, 436, 315, 248, 208, 93, 0
	2	2560, Roi Et	2, 12, 15, 28, 25, 49, 45, 75, 350, 735, 555, 129, 61, 38, 29, 16, 0
Blind Test	2	2562, Trat	0, 4, 3, 5, 7, 9, 21, 11, 58, 94, 108, 90, 43, 20, 15, 6, 0
	2	2562, Narathiwat	0, 3, 9, 4, 7, 8, 18, 20, 63, 113, 147, 115, 56, 30, 18, 13, 0
Training	3	2558, Chumphon	0, 3, 2, 10, 8, 6, 6, 16, 50, 125, 124, 68, 34, 20, 19, 5, 0
	3	2557, Mae Hong Son	0, 0, 1, 0, 3, 5, 9, 15, 42, 77, 117, 62, 34, 36, 16, 9, 0
Blind Test	3	2562, Chai Nat	0, 0, 2, 1, 1, 2, 8, 5, 28, 58, 63, 27, 21, 11, 11, 3, 0
	3	2562, Krabi	0, 7, 2, 12, 10, 3, 6, 8, 38, 61, 64, 30, 26, 13, 4, 5, 0
Training	4	2561, Ratchaburi	0, 9, 9, 14, 18, 22, 34, 46, 140, 320, 291, 139, 69, 34, 28, 13, 0
	4	2556, Tak	1, 4, 8, 12, 14, 21, 23, 21, 93, 201, 260, 153, 71, 29, 14, 13, 0
Blind Test	4	2562, Loei	0, 12, 21, 14, 22, 26, 25, 38, 145, 309, 305, 127, 55, 35, 35, 19, 0
	4	2562, Nakhon Phanom	0, 4, 1, 3, 10, 17, 20, 25, 90, 221, 149, 62, 52, 23, 18, 9, 0
Training	5	2560, Amnat Charoen	0, 2, 1, 3, 3, 6, 9, 16, 68, 127, 96, 19, 15, 7, 3, 3, 0
	5	2558, Surat Thani	0, 1, 2, 2, 6, 8, 6, 6, 43, 85, 169, 87, 35, 11, 7, 3, 0
Blind Test	5	2562, Amnat Charoen	0, 1, 1, 1, 3, 7, 8, 11, 55, 102, 79, 23, 9, 9, 9, 7, 0

**Table 10:** The example of clustering results from the selected models for the dengue fever dataset for the career category.

Data	Cluster	Province	(Farmers, Public Servant, General Contractor, Merchant, Housekeeper, Student, Military/Police, Fisherman, Teacher, Other, Unknown, Herdsman, Priest, Special Occupation, Public Health Personnel)
Training	1	2561, Phangnga	11, 5, 80, 4, 6, 111, 0, 0, 2, 6, 96, 0, 0, 0, 1
	1	2559, Samut Sakhon	4, 2, 87, 3, 11, 125, 1, 0, 0, 2, 58, 0, 1, 0, 0
Blind Test	1	No data are assigned	
Training	2	2561, Loei	31, 1, 21, 9, 4, 206, 2, 0, 0, 2, 36, 0, 0, 0, 2
	2	2560, Sa Kaeo	12, 0, 43, 5, 5, 262, 4, 0, 0, 0, 38, 0, 2, 0, 0
Blind Test	2	2562, Satun	6, 3, 5, 1, 0, 58, 0, 0, 0, 1, 9, 0, 1, 0, 0
	2	2562, Ang Thong	3, 2, 32, 3, 5, 56, 0, 0, 1, 2, 9, 2, 0, 0, 0
Training	3	2561, Nakhon Ratchasima	87, 24, 279, 38, 62, 1431, 23, 0, 8, 36, 418, 0, 9, 0, 8
	3	2555, Nakhon Ratchasima	41, 13, 194, 20, 30, 963, 14, 0, 3, 9, 193, 0, 4, 0, 3
Blind Test	3	2562, Kalasin	76, 6, 75, 12, 22, 517, 4, 0, 1, 6, 231, 0, 4, 0, 3
	3	2562, Ubon Ratchathani	299, 18, 220, 27, 260, 3610, 16, 0, 7, 24, 1595, 0, 21, 0, 3
Training	4	2560, Phrae	57, 8, 142, 7, 8, 303, 1, 0, 2, 3, 85, 0, 9, 0, 1
	4	2558, Trat	61, 9, 173, 29, 29, 264, 3, 16, 4, 14, 57, 0, 5, 0, 11
Blind Test	4	2562, Narathiwat	57, 12, 120, 6, 31, 311, 25, 0, 9, 6, 41, 0, 0, 0, 6
	4	2562, Yala	148, 11, 78, 22, 50, 459, 26, 0, 14, 16, 85, 0, 0, 0, 1
Training	5	2561, Buri Ram	29, 2, 46, 4, 5, 686, 0, 0, 0, 5, 120, 0, 2, 0, 1
	5	2557, Nakhon Sawan	22, 5, 54, 7, 16, 179, 5, 0, 0, 8, 113, 0, 2, 0, 0
Blind Test	5	No data are assigned	

**Table 11:** The example of clustering results from the selected models for the dengue fever dataset for the month category.

Data	Cluster	Province	(Jan, Feb, Mar, Apr, May, Jun, Jul, Aug, Sep, Oct, Nov, Dec)
Training	1	2561, Nakhon Pathom	57, 41, 43, 79, 114, 222, 247, 251, 206, 246, 256, 228
	1	2556, Rayong	85, 77, 54, 59, 81, 127, 142, 95, 84, 63, 59, 30
Blind Test	1	Pattani	53, 37, 18, 11, 39, 71, 88, 75, 50, 56, 44, 21
	1	Phetchabun	5, 7, 20, 29, 60, 174, 147, 122, 83, 35, 22, 6
Training	2	2559, Chanthaburi	43, 31, 37, 16, 38, 62, 108, 72, 41, 45, 40, 9
	2	2558, Nan	0, 0, 2, 13, 60, 73, 101, 77, 47, 16, 8, 7
Blind Test	2	Phrae	1, 7, 7, 8, 20, 53, 108, 80, 35, 22, 11, 5
	2	Uttaradit	10, 7, 10, 46, 25, 74, 171, 67, 54, 37, 22, 15
Training	3	2561, Surin	10, 5, 19, 23, 145, 277, 380, 273, 257, 117, 49, 49
	3	2556, Mae Hong Son	2, 10, 11, 32, 152, 333, 398, 275, 112, 63, 60, 25
Blind Test	3	Kalasin	13, 17, 32, 43, 50, 172, 203, 183, 98, 69, 56, 21
	3	Phitsanulok	12, 14, 20, 14, 18, 42, 56, 87, 99, 61, 46, 15
Training	4	2560, Maha Sarakham	58, 46, 72, 45, 98, 275, 388, 402, 198, 81, 33, 10
	4	2556, Maha Sarakham	58, 46, 72, 45, 98, 275, 388, 402, 198, 81, 33, 10
Blind Test	4	Maha Sarakham	20, 25, 32, 17, 14, 89, 145, 146, 152, 93, 35, 13
	4	Loei	6, 2, 9, 48, 103, 288, 303, 141, 119, 100, 56, 13
Training	5	2560, Yasothon	13, 14, 11, 22, 65, 107, 84, 86, 44, 16, 5, 1
	5	2556, Yasothon	13, 14, 11, 22, 65, 107, 84, 86, 44, 16, 5, 1
Blind Test	5	Nakhon Phanom	2, 11, 15, 49, 166, 241, 107, 68, 26, 11, 5, 3
	5	Chumphon	30, 38, 36, 47, 64, 69, 77, 53, 45, 35, 11, 11

**Table 12:** The example of clustering results from the selected models for the influenza dataset for the age range category.

Data	Cluster	Province	(<28 Days, <1 Year, 1+, 2+, 3+, 4+, 5+, 6+, 7–9, 10–14, 15–24, 25–34, 35–44, 45–54, 55–64, 65+, Unknown)
Training	1	2559, Chachoengsao	0, 51, 44, 34, 53, 46, 50, 42, 112, 95, 120, 158, 101, 63, 48, 36, 0
	1	2552, Maha Sarakham	0, 2, 7, 9, 12, 5, 6, 16, 55, 128, 200, 80, 61, 46, 48, 16, 3
Blind Test	1	2562, Mae Hong Son	0, 37, 51, 58, 56, 55, 50, 63, 135, 162, 148, 150, 111, 67, 53, 50, 0
	1	2562, Kalasin	0, 41, 52, 62, 70, 77, 70, 78, 210, 261, 182, 169, 108, 129, 94, 83, 0
Training	2	2561, Lop Buri	1, 63, 105, 79, 96, 86, 78, 72, 152, 138, 395, 221, 152, 135, 113, 98, 0
	2	2560, Nakhon Ratchasima	2, 253, 398, 391, 471, 451, 478, 523, 1176, 1370, 2807, 1197, 1314, 1327, 1222, 1198, 0
Blind Test	2	2562, Chiang Mai	4, 435, 765, 847, 864, 963, 1020, 1202, 2844, 2556, 2277, 3521, 2202, 1034, 891, 434, 0
	2	2562, Bangkok	6, 1253, 2936, 2804, 3023, 3459, 3646, 4672, 10564, 10389, 9066, 14720, 11842, 6415, 4266, 3451, 1
Training	3	2561, Phatthalung	1, 89, 112, 120, 105, 110, 101, 60, 166, 172, 90, 112, 128, 99, 89, 81, 0
	3	2558, Phitsanulok	1, 85, 126, 101, 98, 81, 48, 54, 137, 101, 116, 83, 62, 56, 29, 29, 0
Blind Test	3	2562, Phangnga	4, 106, 125, 107, 125, 102, 82, 93, 197, 160, 106, 98, 91, 59, 49, 53, 0
	3	2562, Sukhothai	1, 60, 111, 113, 138, 110, 124, 113, 315, 301, 294, 295, 227, 120, 98, 78, 0
Training	4	2561, Chai Nat	0, 4, 7, 7, 7, 10, 3, 5, 18, 26, 14, 13, 21, 11, 17, 3, 0
	4	2559, Ranong	0, 3, 2, 4, 6, 4, 3, 2, 5, 7, 5, 7, 9, 6, 3, 2, 0
Blind Test	4	2562, Pattani	0, 25, 28, 38, 31, 30, 21, 15, 44, 46, 84, 81, 40, 34, 39, 56, 0
	4	2562, Satun	0, 19, 42, 20, 20, 24, 21, 12, 28, 32, 28, 20, 21, 19, 23, 29, 0

**Table 13:** The example of clustering results from the selected models for the influenza dataset for the career category.

Data	Cluster	Province	(Farmers, Public Servant, General Contractor, Merchant, Housekeeper, Student, Military/Police, Fisherman, Teacher, Other, Unknown, Herdsman, Priest, Special Occupation, Public Health Personnel)
Training	1	2559, Khon Kaen	173, 115, 278, 98, 70, 823, 8, 0, 4, 98, 694, 0, 8, 0, 1
	1	2559, Surat Thani	125, 44, 442, 67, 42, 741, 9, 2, 6, 129, 655, 1, 2, 1, 9
Blind Test	1	2562, Mae Hong Son	107, 29, 234, 10, 18, 425, 7, 0, 2, 4, 393, 0, 2, 0, 15
	1	2562, Mukdahan	237, 22, 220, 5, 0, 925, 41, 0, 4, 15, 588, 1, 11, 0, 0
Training	2	2558, Mae Hong Son	12, 5, 14, 1, 2, 38, 0, 0, 0, 0, 96, 0, 0, 0, 0
	2	2558, Roi Et	18, 13, 13, 0, 1, 66, 3, 0, 0, 0, 64, 0, 0, 0, 0
Blind Test	2	2562, Nakhon Nayok	14, 4, 101, 3, 0, 109, 6, 0, 0, 1, 192, 0, 1, 0, 0
	2	2562, Sing Buri	16, 19, 165, 11, 1, 209, 10, 0, 0, 4, 169, 0, 0, 0, 14
Training	3	2554, Tak	78, 11, 136, 13, 1, 139, 3, 0, 0, 7, 104, 0, 0, 2, 4
	3	2551, Songkhla	119, 14, 81, 10, 11, 58, 4, 1, 0, 1, 74, 0, 0, 0, 1
Blind Test	3	No data are assigned	

**Table 14:** The example of clustering results from the selected models for the influenza dataset for the month category.

Data	Cluster	Province	String. (Jan, Feb, Mar, Apr, May, Jun, Jul, Aug, Sep, Oct, Nov, Dec)
Training	1	2561, Phetchaburi	77, 85, 54, 35, 28, 37, 71, 72, 116, 64, 46, 38
	1	2559, Phichit	74, 263, 294, 42, 15, 39, 37, 75, 267, 293, 92, 42
Blind Test	1	2562, Nong Bua Lam Phu	29, 92, 136, 30, 13, 60, 39, 45, 77, 55, 34, 86
	1	2562, Samut Songkhram	29, 59, 63, 68, 19, 25, 41, 68, 92, 66, 63, 44
Training	2	2561, Nan	183, 112, 123, 86, 76, 136, 126, 244, 450, 221, 95, 63
	2	2558, Samut Prakan	89, 218, 224, 129, 91, 114, 114, 121, 173, 184, 180, 134
Blind Test	2	2562, Tak	90, 145, 209, 84, 71, 235, 203, 455, 565, 288, 259, 94
	2	2562, Uttaradit	182, 467, 437, 90, 61, 169, 193, 465, 803, 394, 242, 173
Training	3	2560, Nakhon Sawan	159, 117, 89, 42, 63, 147, 500, 1038, 999, 618, 275, 151
	3	2560, P.Nakhon S.Ayutthaya	121, 106, 62, 40, 60, 306, 412, 546, 696, 320, 114, 88
Blind Test	3	2562, Narathiwat	250, 135, 117, 41, 37, 67, 120, 160, 263, 269, 168, 211
	3	2562, Phatthalung	190, 285, 178, 49, 25, 77, 67, 92, 409, 264, 258, 188

**Table 15:** The example of clustering results from the selected models for the HBV dataset for the age range category.

Data	Cluster	Province	(<28 Days, <1 Year, 1+, 2+, 3+, 4+, 5+, 6+, 7–9, 10–14, 15–24, 25–34, 35–44, 45–54, 55–64, 65+, Unknown)
Training	1	2559, Si Sa Ket	0, 0, 0, 0, 0, 0, 0, 0, 0, 0, 18, 36, 39, 39, 14, 6, 0
	1	2558, Nakhon Si Thammarat	0, 0, 0, 0, 0, 0, 0, 0, 0, 0, 14, 17, 34, 27, 15, 9, 0
Blind Test	1	2562, Chanthaburi	0, 1, 0, 0, 0, 0, 1, 0, 1, 0, 12, 32, 38, 14, 14, 5, 0
	1	2562, Tak	0, 0, 1, 0, 0, 0, 0, 0, 0, 1, 16, 27, 25, 21, 13, 10, 0
Training	2	2559, Nakhon Phanom	0, 0, 0, 0, 0, 0, 0, 0, 0, 0, 4, 4, 8, 6, 9, 3, 0
	2	2558, Sing Buri	0, 0, 0, 0, 0, 0, 0, 0, 0, 0, 1, 0, 3, 0, 1, 3, 0
Blind Test	2	2562, Ang Thong	0, 0, 0, 0, 0, 0, 0, 0, 0, 0, 1, 1, 0, 0, 2, 1, 0
	2	2562, Bungkan	0, 0, 0, 0, 0, 0, 0, 0, 0, 0, 1, 1, 4, 5, 2, 0, 0
Training	3	2558, Sakon Nakhon	0, 0, 0, 0, 0, 0, 0, 0, 0, 0, 3, 13, 12, 10, 4, 2, 0
	3	2550, Ratchaburi	0, 0, 0, 0, 0, 0, 0, 0, 0, 0, 6, 9, 11, 11, 5, 7, 0
Blind Test	3	2562, Rayong	0, 0, 0, 0, 0, 0, 0, 0, 0, 0, 6, 18, 12, 16, 10, 2, 0
	3	2562, Maha Sarakham	0, 0, 0, 0, 0, 0, 0, 0, 0, 0, 0, 14, 21, 12, 3, 2, 0
Training	4	2549, Nonthaburi	0, 1, 0, 0, 0, 0, 0, 0, 0, 2, 1, 10, 5, 10, 7, 0, 0
	4	2551, Bangkok	0, 0, 1, 0, 0, 0, 0, 4, 1, 2, 38, 52, 30, 30, 9, 9, 0
Blind Test	4	2562, Chiang Mai	0, 0, 0, 1, 1, 1, 0, 0, 1, 2, 17, 44, 45, 40, 27, 10, 0
	4	2562, Nakhon Sawan	0, 0, 0, 0, 0, 0, 0, 0, 1, 0, 2, 8, 3, 6, 3, 5, 0
Training	5	2557, Chachoengsao	0, 0, 0, 0, 0, 0, 0, 0, 0, 0, 9, 29, 41, 44, 23, 11, 0
	5	2555, Loei	0, 0, 0, 0, 0, 0, 0, 0, 0, 0, 12, 20, 22, 27, 12, 8, 0
Blind Test	5	2562, Chai Nat	0, 0, 0, 0, 0, 0, 0, 0, 0, 0, 1, 2, 2, 4, 1, 3, 0
	5	2562, Chon Buri	0, 0, 0, 0, 0, 0, 0, 0, 0, 0, 8, 32, 34, 18, 15, 9, 0
Training	6	2558, Phatthalung	0, 0, 0, 0, 0, 0, 0, 0, 0, 0, 1, 2, 7, 12, 3, 2, 0
	6	2552, Chon Buri	0, 0, 0, 0, 0, 0, 0, 0, 0, 0, 13, 6, 11, 4, 6, 2, 0
Blind Test	6	No data are assigned	

**Table 16:** The example of clustering results from the selected models for the HBV dataset for the career category.

Data	Cluster	Province	(Farmers, Public Servant, General Contractor, Merchant, Housekeeper, Student, Military/Police, Fisherman, Teacher, Other, Unknown, Herdsman, Priest, Special Occupation, Public Health Personnel)
Training	1	2561, Phetchabun	128, 1, 193, 4, 8, 14, 19, 0, 1, 1, 63, 0, 4, 0, 0
	1	2549, Udon Thani	51, 3, 17, 4, 5, 8, 0, 0, 0, 3, 10, 0, 0, 0, 0
Blind Test	1	2562, Chiang Rai	53, 2, 103, 4, 6, 5, 1, 0, 0, 0, 11, 0, 3, 0, 1
	1	2562, Nakhon Si Thammarat	55, 0, 61, 1, 0, 5, 2, 1, 0, 1, 42, 0, 0, 0, 0
Training	2	2561, Nonthaburi	0, 0, 14, 1, 0, 2, 0, 0, 0, 0, 1, 0, 0, 0, 0
	2	2559, Nong Khai	8, 7, 26, 2, 0, 2, 0, 0, 0, 0, 6, 0, 0, 0, 0
Blind Test	2	2562, Suphan Buri	6, 1, 9, 1, 0, 1, 0, 0, 0, 0, 0, 0, 0, 0, 0
	2	2562, Bungkan	7, 1, 4, 0, 0, 0, 0, 0, 0, 0, 1, 0, 0, 0, 0

**Table 17:** The example of clustering results from the selected models for the HBV dataset for the month category.

Data	Cluster	Province	(Jan, Feb, Mar, Apr, May, Jun, Jul, Aug, Sep, Oct, Nov, Dec)
Training	1	2561, Lamphun	1, 0, 0, 4, 1, 1, 0, 1, 2, 1, 0, 2
	1	2560, Phuket	2, 0, 2, 3, 0, 1, 3, 2, 2, 2, 0, 2
Blind Test	1	2562, Lamphun	3, 1, 1, 2, 0, 0, 1, 3, 4, 0, 2, 2
	1	2562, Narathiwat	0, 2, 1, 1, 4, 2, 7, 2, 3, 0, 5, 3
Training	2	2561, Chiang Mai	55, 28, 24, 17, 15, 23, 23, 17, 11, 16, 21, 13
	2	2559, Loei	22, 26, 24, 19, 17, 11, 19, 13, 5, 10, 14, 19
Blind Test	2	2562, Si Sa Ket	29, 15, 15, 12, 21, 23, 10, 13, 13, 16, 18, 11
	2	2562, Surat Thani	17, 16, 15, 15, 16, 9, 12, 17, 15, 10, 17, 11
Training	3	2561, Phrae	3, 2, 3, 2, 1, 2, 3, 2, 3, 3, 2, 1
	3	2559, Phichit	11, 6, 7, 9, 9, 5, 6, 2, 9, 5, 9, 2
Blind Test	3	2562, Nakhon Sawan	0, 1, 1, 4, 1, 2, 4, 5, 4, 1, 4, 1
	3	2562, Phichit	1, 4, 6, 6, 5, 8, 1, 1, 1, 3, 2, 4

## Data Availability

Not applicable.
